# The role and prognostic value of PANoptosis-related genes in skin cutaneous melanoma

**DOI:** 10.3389/fimmu.2025.1605977

**Published:** 2025-06-06

**Authors:** Huijing Feng, Linzi Jia, Yanan Ma, Pengmin Liu, Xiaoling Yang, Lina Hu, Kai Xu, Fan Yang, Dongfeng Zhang, Jian Li, Qi Mei, Fei Han

**Affiliations:** ^1^ Cancer Center, Shanxi Bethune Hospital, Shanxi Academy of Medical Sciences, Tongji Shanxi Hospital, Third Hospital of Shanxi Medical University, Taiyuan, Shanxi, China; ^2^ Department of General Medicine, Shanxi Province Cancer Hospital, Taiyuan, Shanxi, China; ^3^ Department of Pathology, Shanxi Bethune Hospital, Shanxi Academy of Medical Sciences, Tongji Shanxi Hospital, Third Hospital of Shanxi Medical University, Taiyuan, Shanxi, China; ^4^ Department of Dermatology and Venereology, Shanxi Bethune Hospital, Shanxi Academy of Medical Sciences, Tongji Shanxi Hospital, Third Hospital of Shanxi Medical University, Taiyuan, Shanxi, China; ^5^ Department of Bone and Soft-Tissue Tumor, Shanxi Province Cancer Hospital, Taiyuan, Shanxi, China; ^6^ Department of Thoracic Oncology, Linfen Central Hospital, Linfen, Shanxi, China; ^7^ Institute of Molecular Medicine and Experimental Immunology (IMMEI), University Hospital Bonn, Bonn, North Rhine - Westphalia, Germany; ^8^ Department of Head and Neck Surgery, Shanxi Province Cancer Hospital/Shanxi Hospital Affiliated to Cancer Hospital, Chinese Academy of Medical Sciences/Cancer Hospital Affiliated to Shanxi Medical University, Taiyuan, Shanxi, China

**Keywords:** skin cutaneous melanoma, PANoptosis, prognosis, competitive endogenous RNA, PANoptosis-related genes

## Abstract

**Introduction:**

Skin cutaneous melanoma (SKCM), a malignant tumor, has PANoptosis implicated in its progression and metastasis. However, the exact mechanisms remain unclear. This study aims to develop a prognostic model for SKCM based on PANoptosis.

**Methods:**

SKCM - related datasets were retrieved from public databases. Differentially expressed PANoptosis - related genes (DEPRGs) were determined by intersecting differentially expressed genes from differential expression analysis and key module genes from weighted gene co - expression network analysis (WGCNA). Prognostic genes for SKCM were derived using Cox analysis and machine learning algorithms, leading to the construction and validation of a prognostic model. Independent prognostic factors were identified, and a nomogram was developed. Enrichment analysis and immune infiltration analysis were performed for the two risk groups. A competitive endogenous RNA (ceRNA) network was constructed, and potential therapeutic drugs were predicted. Bioinformatics findings were validated experimentally using reverse transcription quantitative PCR (RT - qPCR).

**Results:**

CD8A, ADAMDEC1, CD69, CRIP1, LSP1, BCL11B, and CCR7 were identified as prognostic genes. The risk model and nomogram showed excellent predictive abilities for SKCM patients. Genes in both high - and low - risk groups were linked to cytokine - regulated immune responses, with nine differential immune cells identified between the groups. The ceRNA network revealed that prognostic genes were regulated by several miRNAs (such as hsa-miR-330-5p) and lncRNAs (such as AL355075.4). MPPG and DT - 1687, associated with LSP1, may offer promising treatment options. RT - qPCR validation confirmed significant expression differences of CD8A, ADAMDEC1, CD69, CRIP1, and BCL11B between SKCM and control samples.

**Discussion:**

This study presents a robust prognostic model for SKCM based on PANoptosis - related genes, providing a theoretical foundation for SKCM treatment.

## 1 Introduction

Skin cutaneous melanoma (SKCM) is a malignant skin tumor resulting from the transformation of melanocytes in the epidermal basal layer (1, 2). The incidence of SKCM in the United States is approximately 31.91 per 100,000, compared to 0.5 per 100,000 in China (3), accounting for 0.7% of all cancer-related deaths (4). Current treatment strategies for SKCM involve a multimodal approach, including surgery, radiotherapy, chemotherapy, targeted therapy, and immunotherapy (5). However, SKCM has a high propensity for metastasis, with many patients diagnosed at advanced stages. Stage IV patients have a poor prognosis, with a 5-year survival rate of less than 5% (6). Therefore, identifying novel prognostic biomarkers is crucial for improving treatment outcomes. PANoptosis is an inflammatory programmed cell death pathway controlled by the PANoptosome complex, which integrates key features of Pyroptosis, Apoptosis, and/or Necroptosis (7, 8). PANoptosis plays a significant role in cancer biology and therapeutic strategies. Preclinical studies have shown that IRF1-dependent PANoptosis inhibits colorectal cancer progression in murine models, and combined TNF-α and IFN-γ treatment induces PANoptotic cell death in human cancer cells (9, 10). Recent research has highlighted the prognostic index (PANGPI) subtype system, constructed using PANoptosis-related genes, as a predictor of prognosis and immunotherapy response in diffuse large B-cell lymphoma (DLBCL), offering insights into personalized treatment (11). Similarly, in patients with ovarian cancer (OC), prognostic models based on PANoptosis genes effectively predict both prognosis and immune responses (12). Moreover, PANoptosis plays a critical role in programmed cell death, cancer progression, and immune evasion in melanoma, presenting new avenues for personalized treatment (13, 14). Overall, the prognostic and immune implications of PANoptosis in various cancers provide valuable guidance for individualized treatment approaches. The PANoptosis gene signature consists of 27 genes, including cytosolic sensors, adaptors, effector proteins, and upstream regulatory components. Tumor specimens can be stratified into PANoptosis-High and PANoptosis-Low cohorts based on gene expression profiles. The PANoptosis-High cluster correlates with significantly improved overall survival (OS) in SKCM. Furthermore, the expression differences between PANoptosis clusters are linked to key molecular mechanisms, such as the activation of proliferation pathways, aneuploidy, immune cell density and activation, and the regulation of barrier genes. These findings contribute to stratifying patient prognosis based on the PANoptosis phenotype and offer a new perspective for personalized SKCM treatment (14). However, the clinical prognostic value of PANoptosis-related genes in SKCM requires further investigation.

This study explored the role of PANoptosis in SKCM and developed a novel prognostic model based on PANoptosis-related genes. Additionally, differences in immune cell infiltration and their correlation with prognostic models were investigated. Overall, this study provides a theoretical foundation for SKCM treatment and guides the selection of optimal therapeutic strategies.

## 2 Materials and methods

### 2.1 Data sources

Transcriptome data related to SKCM were retrieved from The Cancer Genome Atlas (TCGA) database (https://portal.gdc.cancer.gov/). A total of 477 SKCM samples were obtained from the TCGA dataset. After samples lacking survival information were removed, 457 samples with complete survival information were retained for subsequent analysis. Clinical information samples, including age, gender, tumor stage, M, N, and T staging, were extracted. After samples lacking clinical information were deleted, 236 samples remained for subsequent clinical information related analyses.

Additionally, datasets GSE46517 and GSE65904 were obtained from the Gene Expression Omnibus (GEO) database (https://www.ncbi.nlm.nih.gov/geo/). The GSE46517 dataset contained 121 samples, which included metastatic melanoma, primary melanoma, nevus, and normal skin samples. The metastatic melanoma, melanoma, and nevus samples were excluded, and 31 primary SKCM samples along with 7 normal samples were retained for analysis. The GSE65904 dataset contained 210 SKCM samples, and all of them were included in the subsequent analysis. A total of 19 PANoptosis-related genes (PRGs) were sourced from existing literature (**Supplementary Table 1**) (15).

### 2.2 Calculation of PANoptosis scores and identification of key module genes

In TCGA-SKCM dataset, the enrichment scores of the PRGs in the SKCM samples were calculated using the ssGSEA method in the GSVA package (version 1.42.0) of R language. The parameters were set as gsva(as.matrix(exp), geneSet, method=‘ssgsea’, kcdf=‘Gaussian’, abs.ranking=TRUE). Subsequently, the enrichment scores of PRGs were defined as the PRGs score. To explore the impact of the PANoptosis score on the survival of SKCM patients, SKCM patients were divided into a high-PRG-score cohort and a low-PRG-score cohort based on the median PANoptosis score (16). This approach effectively balanced the sample sizes and provided a stable statistical foundation for subsequent survival analysis. Kaplan-Meier (K-M) survival analysis was then performed to compare survival rates between the two groups (p < 0.05) (17). Differentially expressed genes (DEGs) between the two groups were identified using the DEseq2 package in R (|log_2_FC| > 1 and P adj. < 0.05) (18) Volcano and heat maps were generated using the ggplot2 and pheatmap packages in R, respectively, to visualize the results (19).

To identify key PANoptosis-associated module genes in SKCM samples, weighted gene co-expression network analysis (WGCNA) was conducted. Firstly, outlier samples in the TCGA-SKCM dataset were removed through cluster analysis. Then, the coefficient of determination (R^^2^) of the scale-free network was set to 0.85, and the optimal soft threshold was obtained when (R^^2^) first exceeded the critical value of 0.85 and the average connectivity of the co-expression network was close to 0 (20). Next, according to the screened soft threshold, a scale-free network was constructed, and the minimum number of genes in each module was set to 200, so that genes could be effectively grouped into multiple modules. Subsequently, the PANoptosis score was taken as the trait. Through Pearson correlation analysis of the correlation between the module and the trait, the module with the strongest significant correlation was selected as the key module.^20^ Genes with |Module Membership (MM)| > 0.8 and |Gene Significance (GS)| > 0.5 in the key module were considered as key module genes (21, 22).

### 2.3 Identification and enrichment analyses of differentially expressed PRGs

To identify DEGs in samples from normal and SKCM cohorts, the limma R package was used with the GSE46517 dataset (|log_2_FC| > 1 and P adj. < 0.05) (23). A volcano plot was created using the ggplot2 R package (version 3.3.6) to illustrate the filtering results (19). Heatmaps of DEG expression were visualized using the pheatmap R package (version 1.0.12) (23). Differentially expressed PANoptosis-related genes (DEPRGs) were identified by intersecting DEGs between normal and SKCM samples, key module genes, and DEGs from high and low-PANoptosis score groups.

Finally, Kyoto Encyclopedia of Genes and Genomes and Gene Ontology (GO) analyses for the DEPRGs were performed using the clusterProfiler R package (version 4.4.4) (24).

### 2.4 Developing a prognostic model for patients with SKCM

In the TCGA-SKCM dataset, 457 samples were divided into a validation set (137 samples) and a training dataset (320 samples). Univariate Cox analysis was performed on the DEPRGs in the training dataset to identify prognostic-related genes (HR ≠ 1, p < 0.05). Subsequently, the least absolute shrinkage and selection operator (Lasso) was applied using the R package glmnet. Based on 10-fold cross-validation, prognostic-related genes with the smallest cross-validation error value (λ value) and non-zero regression coefficients were selected as the characteristic genes for SKCM patients. A multifactorial Cox analysis was then conducted on these characteristic genes to identify prognostic genes and build a prognostic model for SKCM (25). The variance inflation factor (VIF) values of the multivariate Cox regression model were calculated using the vif function, and the model performance metric, the C-index, was computed (C-index > 0.6). Subsequently, the proportional hazards (PH) assumption of the multivariate Cox regression model was tested using cox.zph (p > 0.05). To investigate whether severe multicollinearity existed among the prognostic genes, a linear model was constructed using the `lm` function with each prognostic gene as the dependent variable and the other genes as independent variables. The VIF for each gene was then calculated using the vif() function from the `car` package. A VIF value less than 5 indicated the absence of severe multicollinearity.

To evaluate the prognostic value of the model, patients with SKCM in the training dataset were classified into high- and low-risk groups based on the median risk score (25). K-M survival analysis was performed to compare survival rates between the two groups. Receiver operating characteristic (ROC) curves for the prognostic model were generated using the survival ROC R package (17). The robustness of the model was further validated using the external GSE65904 dataset and the TCGA-SKCM validation set.

### 2.5 Construction of the nomogram

The independent prognostic value of clinicopathological parameters (including age, history of neoadjuvant treatment, prior treatment.diagnoses, prior systemic therapy, M stage, N stage, T stage, gender and tumor_stage) and risk scores was assessed using univariate and multivariate Cox proportional hazards regression analyses in the training set (26, 27). A nomogram model was then constructed by integrating factors with independent prognostic significance (17). The rms package (version 6.5.1) was used to generate a calibration curve, which was applied to assess the precision of the nomogram’s prognostic ability (28).

### 2.6 Gene set enrichment analyses

To explore the biological functions and pathways associated with the prognostic genes in SKCM, GSEA was performed using TCGA-SKCM data. The DESeq2 R package was used to identify differential gene expression between the high- and low-risk groups in the training dataset. These genes were ranked according to their log_2_ fold-change (log_2_FC) values and subjected to GSEA through the clusterProfiler and org.Hs.eg.db R packages (24).

### 2.7 Estimation of the immune microenvironment

To examine differences in immune cell infiltration between the high- and low-risk cohorts, the CIBERSORT algorithm was applied to determine the distribution of 22 immune cell types in TCGA-SKCM (p < 0.05). A bar plot, created with the ggplot2 R package, was used to visualize the immune cell expression in the two risk groups (25). The Wilcoxon test was employed to compare the distribution differences of immune cells between the groups (p < 0.05) (29). Finally, the psych R package (version 2.3.12) was used to explore the relationships between differentially infiltrated immune cells and prognostic genes through Spearman correlation analysis (30).

### 2.8 Construction of competitive endogenous RNA network

The molecular regulatory mechanisms of prognostic genes were explored in future studies. The miRNAs associated with prognostic genes were identified using the miRTarBase database, and the upstream miRNAs with a connectivity greater than 1 and supported by experimental results were screened (31). Next, miRNA-associated long non-coding RNAs (lncRNAs) were predicted through the starBase database, applying a screening criterion of clipExpNum > 10 (27). Competitive endogenous RNA (ceRNA) networks were constructed using Cytoscape (31).

### 2.9 Predicting potential drugs for the treatment of patients with SKCM

To explore drugs associated with prognostic genes and identify potential treatments for SKCM, potential therapeutic agents for SKCM were predicted using the Drug-Gene Interaction Database (DGIdb) (http://dgidb.genome.wustl.edu/), PubChem (https://pubchem.ncbi.nlm.nih.gov/), and the Therapeutic Target Database (TTD) (https://db.idrblab.net/ttd/), based on the prognostic genes (32–34). The drug-prognostic gene network was then visualized using Cytoscape.

### 2.10 RNA extraction and reverse transcription-quantitative PCR

In this study, RT-qPCR analysis was performed on HSF, MV3, SK-MEL-28, and WM-115 cell lines purchased from the ATCC database when cell confluence reached 70%-90%. Three samples were selected for each cell type. Total RNA was extracted from these cells using TRIzol reagent (Ambion, Austin, USA), and incubated for 15 minutes. One microliter of RNA solution was measured using the NanoPhotometer N50. Reverse transcription to synthesize complementary DNA (cDNA) was performed using the SureScript First-strand cDNA Synthesis Kit (Servicebio, Wuhan, China). Following cDNA synthesis, the solution was diluted 5 to 20 times with RNase/DNase-free distilled water (ddH_2_O). A 10 μl reaction system was configured for qPCR, which contained 3 μl of cDNA, 5 μl of 2xUniversal Blue SYBR Green qPCR Master Mix, 1 μl of 10 μM Forward primer, and 1 μl of 10 μM Reverse primer. RT-qPCR amplification was carried out using a CFX96 Real-Time Fluorescence Quantitative PCR Instrument (Bio-Rad, California, USA) for a total of 40 cycles. Finally, Ct values of prognostic genes and calibrator gene Glyceraldehyde-3-Phosphate Dehydrogenase(GAPDH) were employed to compute relative expression of every prognostic gene in each sample by 2^−△△Ct^ method. The t-test of Graphpad Prism 5 (v 8.0) software (35). GAPDH was used to assess RT-qPCR data (p < 0.05). Detailed information about the RT-qPCR reaction system, primer sequences, and amplification conditions is provided in **Supplementary Tables 2–4**.

### 2.11 Statistical analysis

Statistical analyses were performed using R software (v 4.1.0), and the Wilcoxon test was used to compare pairs within cohorts. A p-value of < 0.05 was considered statistically significant.

### 2.12 Ethics approval and consent to participate

This study, titled The role and prognostic value of PANoptosis-related genes in skin cutaneous melanoma, has been approved by the ethics committee of Shanxi Bethune Hospital. The approval number and date of approval are as follows: [YXLL-2023-087] and [2023-4-11].

## 3 Results

### 3.1 A total of 1460 DEGs and 426 key module genes were identified in TCGA-SKCM

**Figure 1:**
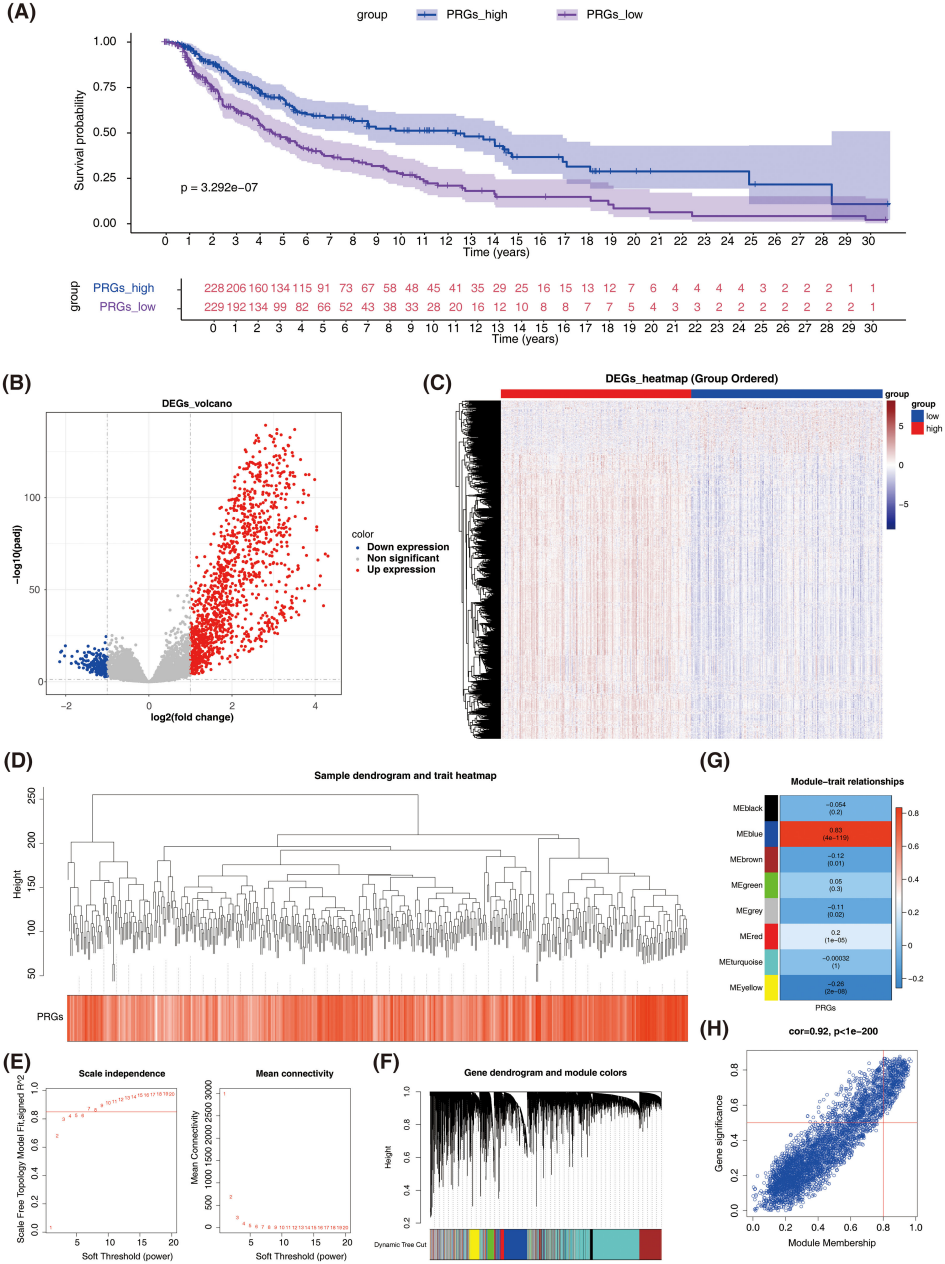
Identification of differentially expressed genes (DEGs) and key module genes related to PANoptosis in SKCM. **(A)** Kaplan-Meier survival analysis comparing overall survival between high and low PANoptosis score groups in TCGA-SKCM dataset. **(B)** Volcano plot showing differentially expressed genes (DEGs) between high and low PANoptosis score groups. Red dots represent up-regulated genes, blue dots represent down-regulated genes, and gray dots represent non-significant genes. **(C)** Heatmap of DEGs expression between high and low PANoptosis score groups. The color scale represents the expression level of genes, with red indicating high expression and blue indicating low expression. **(D)** Sample dendrogram and trait heatmap. **(E)** Screening with soft threshold. Left panel shows the scale independence plot, and the right panel shows the mean connectivity plot. **(F)** Identification of co-expression modules. **(G)** Correlation heatmap. **(H)** Scatter plot of gene significance (GS) versus module membership (MM) for genes in the MEblue module.

In the TCGA-SKCM dataset, individuals in the low PANoptosis score group exhibited significantly lower survival probabilities compared to the high PANoptosis score group (**Figure 1A**). Differential expression analysis revealed 1460 DEGs between the high and low PANoptosis score groups (|log_2_FC| > 1 and P adj. < 0.05), including 219 down-regulated genes and 1241 up-regulated genes (**Figures 1B, C**). All samples in the TCGA-SKCM dataset were successfully clustered, with no outliers observed (**Figure 1D**). To construct a co-expression network and identify modules, the soft threshold power of seven was selected to compute adjacencies (**Figure 1E**). Dynamic tree-cutting identified eight modules (**Figure 1F**), with the MEblue module showing the strongest correlation with PANoptosis scores (cor = 0.83, p < 0.001) (**Figure 1G**). Of the 3801 genes in the MEblue module, 426 were selected as key module genes (**Figure 1H**).

### 3.2 DEPRGs were involved mainly in cytokine and chemokine-mediated cellular immune responses

**Figure 2:**
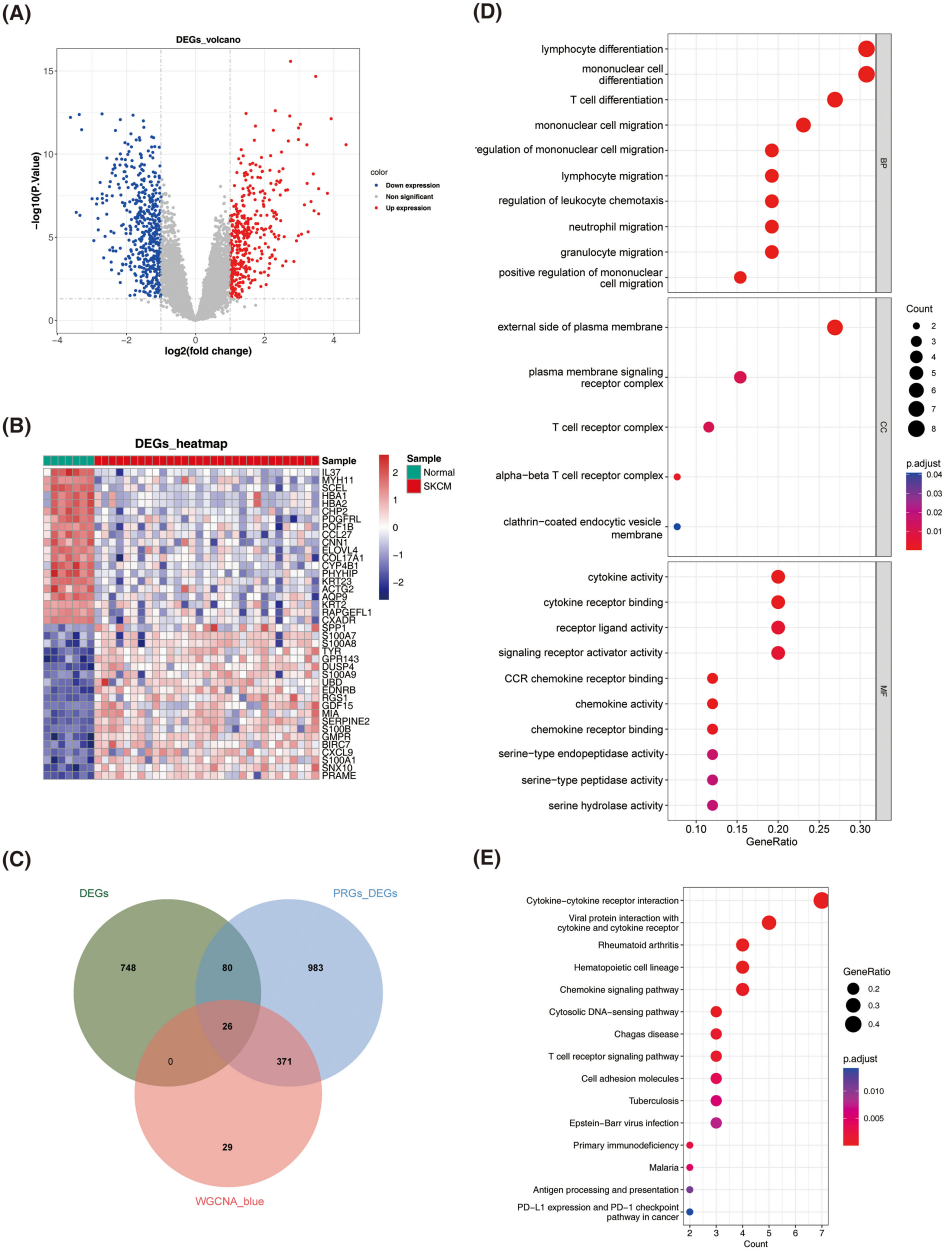
Identification and enrichment analysis of differentially expressed PANoptosis-related genes (DEPRGs) in SKCM. **(A)** Volcano plot of DEGs between normal and SKCM samples. Red dots represent up-regulated genes, blue dots represent down-regulated genes, and gray dots represent non-significant genes. **(B)** Heatmap of DEGs between normal and SKCM samples. The color scale represents the expression level of genes, with red indicating high expression and blue indicating low expression. **(C)** Identification of DEPRGs. **(D)** Dot plot of Gene Ontology (GO) enrichment analysis for DEPRGs. The size of the dots represents the count of genes, and the color represents the adjusted p-value. **(E)** Dot plot of Kyoto Encyclopedia of Genes and Genomes (KEGG) pathway enrichment analysis for DEPRGs. The size of the dots represents the gene ratio, and the color represents the adjusted p-value.

Variable analysis of the GSE46517 dataset identified 854 DEGs between normal and SKCM samples (P adj. < 0.05 and |log_2_FC| > 1), comprising 472 down-regulated genes and 382 up-regulated genes (**Figures 2A, B**).

A total of 26 DEPRGs were identified by intersecting 854 DEGs from normal vs. SKCM samples, 1460 DEGs from high and low PANoptosis score groups, and 426 key module genes (**Figure 2C**). GO analysis revealed that DEPRGs were significantly enriched in lymphocyte differentiation, monocyte differentiation and migration, cytokine activity, and chemokine activity (**Figure 2D**).

KEGG analysis revealed significant enrichment of DEPRGs in cytokine and chemokine signaling pathways, as well as their corresponding receptors (**Figure 2E**).

### 3.3 The prognostic model constructed based on DEPRGs could accurately measure the prognosis of patients with GC

**Figure 3:**
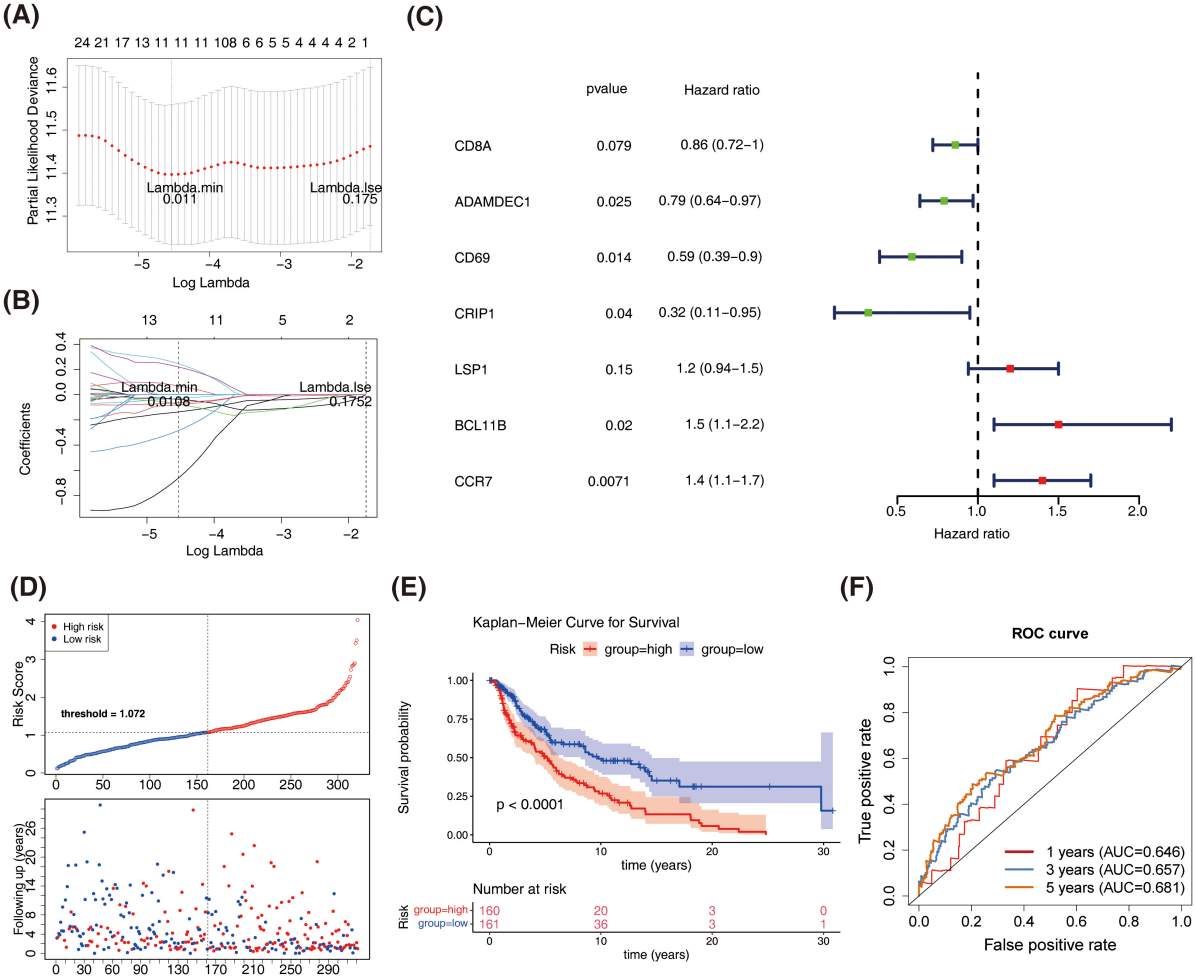
Construction and validation of the prognostic model based on DEPRGs in SKCM. **(A)** LASSO regression analysis for feature selection. **(B)** LASSO coefficient profiles of the 11 characteristic genes. **(C)** Forest plot of multivariate Cox regression analysis. **(D)** Risk score distribution and survival status of SKCM patients in the training dataset. **(E)** Kaplan-Meier survival curves comparing overall survival between high-risk and low-risk groups in the training dataset. **(F)** Receiver operating characteristic (ROC) curves for the prognostic model in the training dataset.

Univariate Cox analysis identified 26 prognosis-related genes in the training dataset (HR ≠ 1, p-value < 0.05) (**Table 1**). Following this, LASSO analysis showed that when the minimum λ value (lambda.min = 0.011) was reached, the minimum error was obtained, and 11 characteristic genes with non-zero regression coefficients were derived (**Figures 3A, B**, **Table 2**). Multifactorial Cox analysis further identified seven prognostic genes and constructed a prognostic model (**Figure 3C**). The VIF value of each gene was less than 5 (**Table 3**), and the C-index of the model was 0.64. The PH assumption test showed that the p-values of all genes were greater than 0.05 (**Table 4**), indicating that these genes had a certain predictive ability. The seven prognostic genes were *CD8A*, *ADAMDEC1*, *CD69*, *CRIP1*, *LSP1*, *BCL11B*, and *CCR7*. The multicollinearity test for the prognostic genes showed that the VIF value for each gene was less than 5, indicating that there was no significant multicollinearity (**Supplementary Figure 1**). Patients in the high-risk cohort, who exhibited shorter survival times, were predominantly found in the DEPRGs cases (**Figure 3D**). The high-risk group demonstrated significantly worse survival outcomes (P < 0.0001) (**Figure 3E**). The prognostic model proved to be reliable in assessing DEPRGs in the training dataset (**Figure 3F**).

**Table 1:** uniCox of result.

Id	z	HR	HR.95L	HR.95H	Pvalue
CCL4	-4.326538547	0.765112517	0.677722246	0.863771504	1.51E-05
CD8A	-4.233557446	0.811290771	0.736425784	0.893766527	2.30E-05
GZMA	-4.136954347	0.820911558	0.747640088	0.901363901	3.52E-05
CD2	-3.971270689	0.833329432	0.76161755	0.911793514	7.15E-05
CTSS	-3.94765638	0.811428873	0.73146643	0.900132651	7.89E-05
IGSF6	-3.885201067	0.749811677	0.648439475	0.867031656	0.000102245
ADAMDEC1	-3.884118128	0.788745277	0.699729299	0.889085412	0.000102702
CCL5	-3.85373029	0.862902052	0.800556964	0.930102396	0.000116332
CYTIP	-3.827594698	0.758701076	0.658657315	0.873940529	0.000129402
CMAHP	-3.820099809	0.539588555	0.393181594	0.740512304	0.000133398
CD69	-3.786170974	0.680986022	0.558161489	0.830838335	0.000152986
CXCL13	-3.780336597	0.853882614	0.786738988	0.92675656	0.000156616
GZMK	-3.697710997	0.812740003	0.728151401	0.907155177	0.000217552
SLAMF8	-3.688843374	0.808352585	0.721949985	0.905095803	0.000225276
PIM2	-3.684370149	0.77763437	0.68025473	0.888954073	0.000229269
CD3G	-3.683302853	0.722203422	0.607364314	0.858756057	0.000230231
CD247	-3.6694957	0.7390696	0.628850034	0.868607527	0.000243029
ITGAL	-3.546744016	0.818890464	0.733286324	0.914488066	0.000390023
IL18	-3.464908909	0.762947709	0.654676337	0.889125165	0.000530411
CRIP1	-3.413927586	0.32188031	0.167902712	0.617065279	0.000640336
LSP1	-2.896506019	0.842419146	0.750128721	0.946064319	0.003773434
IL7R	-2.882973087	0.81372222	0.707317148	0.93613431	0.003939411
LTB	-2.82474386	0.868002408	0.78679817	0.957587612	0.004731843
PLAC8	-2.380909342	0.760242386	0.606669079	0.952691517	0.017269962
BCL11B	-2.272850056	0.775785763	0.623249619	0.965654099	0.023035217
CCR7	-2.125476184	0.878943089	0.780342613	0.990002264	0.033546899

**Table 2:** LASSO of result.

Gene	Regression coefficient
CCL4	-0.036309923
CD8A	-0.079678751
GZMA	0
CD2	0
CTSS	-0.029515353
IGSF6	0
ADAMDEC1	-0.136740393
CCL5	0
CYTIP	0
CMAHP	-0.074567734
CD69	-0.284607168
CXCL13	0
GZMK	0
SLAMF8	0
PIM2	-0.063706757
CD3G	0
CD247	0
ITGAL	0
IL18	0
CRIP1	-0.662353571
LSP1	0.0700072975867431
IL7R	0
LTB	0
PLAC8	0
BCL11B	0.2458603688509
CCR7	0.220594896298752

**Table 3:** The VIF values of the multivariate Cox regression.

Gene	VIF
CD8A	3.03131006000874
ADAMDEC1	2.95042297591821
CD69	4.7734142488617
CRIP1	2.97646297241868
LSP1	3.25516923407163
BCL11B	2.94852743575457
CCR7	4.20569712916761

**Table 4:** PH assumption test.

Gene	P
CD8A	0.245277518691229
ADAMDEC1	0.51221251220946
CD69	0.279092800562971
CRIP1	0.180995978758048
LSP1	0.152248823288871
BCL11B	0.239207649688264
CCR7	0.0885880421429388
GLOBAL	0.0631386724075474

**Figure 4:**
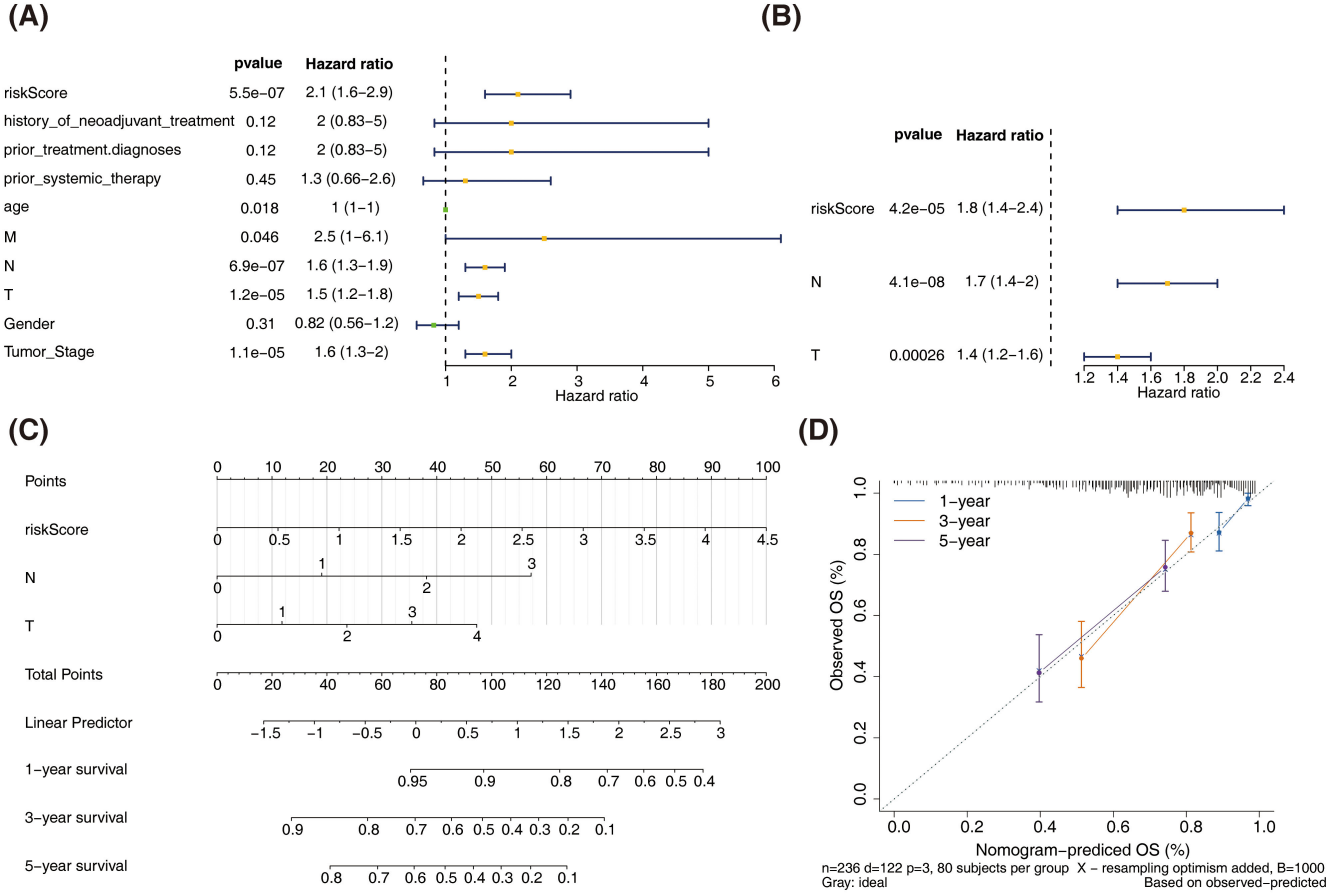
Validation of the prognostic model in the test and external validation datasets. **(A)** Kaplan-Meier survival curves comparing overall survival between high-risk and low-risk groups in the test dataset. **(B)** Kaplan-Meier survival curves comparing overall survival between high-risk and low-risk groups in the external validation dataset (GSE65904). **(C)** Receiver operating characteristic (ROC) curves for the prognostic model in the test dataset. **(D)** Receiver operating characteristic (ROC) curves for the prognostic model in the external validation dataset (GSE65904).

Similar results were obtained in both the GSE65904 dataset and the validation set, with patients in the high-risk group showing slower survival (**Figures 4A, B**). The 1-year, 3-year, and 5-year ROC curves for SKCM patients were plotted. The AUC values were found to be 0.646, 0.657, and 0.681 in GSE65904 set, respectively, and 0.625, 0.660, and 0.646 in validation set, respectively (**Figures 4C, D**). The above results demonstrate that the prognostic model is reliable in evaluating SKCM.

### 3.4 The nomogram model could reliably predict the prognosis of patients with SKCM

**Figure 5:**
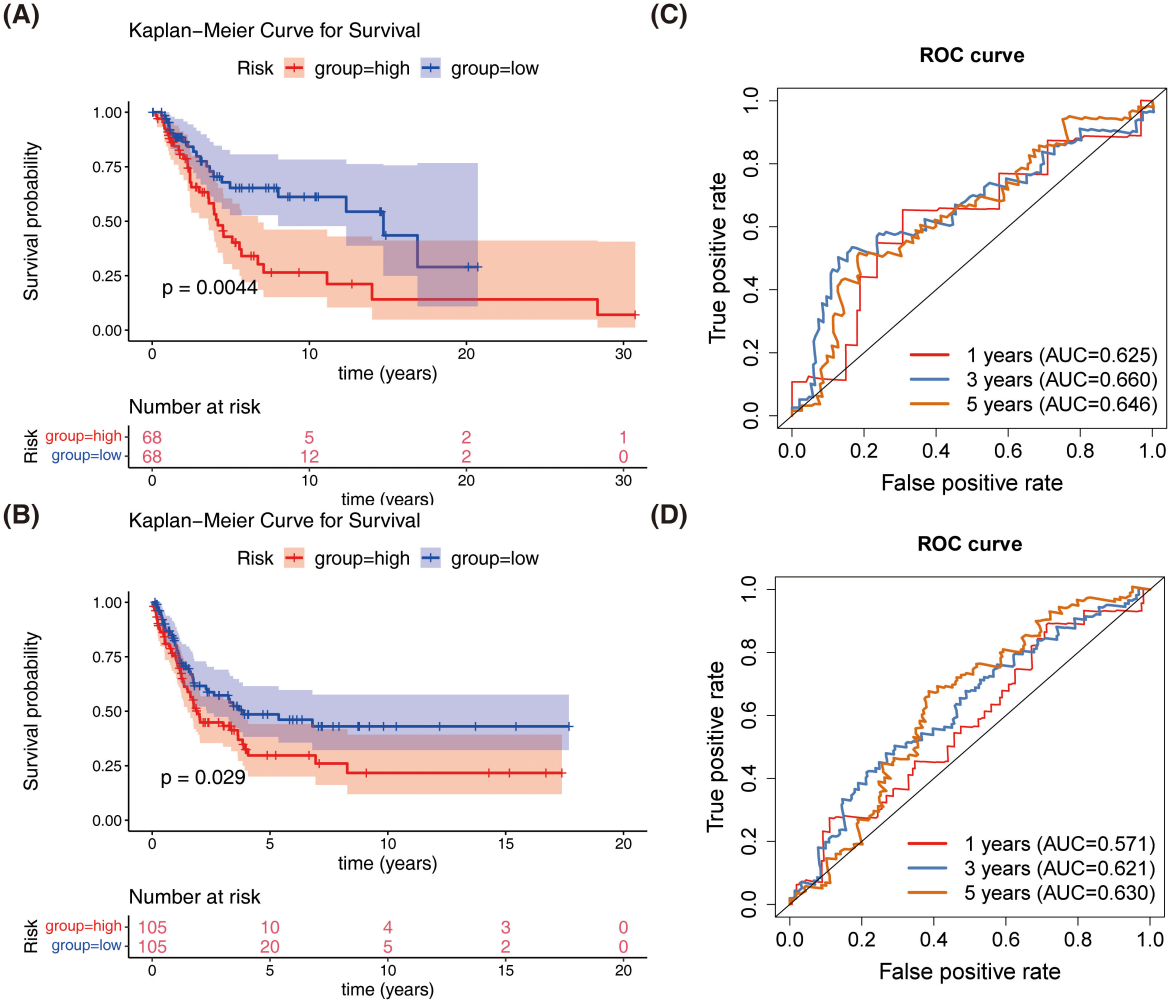
Construction and validation of the nomogram for predicting SKCM patient survival. **(A)** Univariate Cox regression analysis of clinicopathological parameters and risk score in the training dataset. **(B)** Multivariate Cox regression analysis of clinicopathological parameters and risk score in the training dataset. **(C)** Nomogram for predicting 1-year, 3-year, and 5-year overall survival (OS) in SKCM patients. **(D)** Calibration curves for the nomogram predicting 1-year, 3-year, and 5-year OS.

In the training set, univariate Cox analysis revealed significant associations between risk score, age, TNM stage, and clinical stage with the survival of patients with SKCM (P < 0.05). While, history of neoadjuvant treatment, prior treatment diagnoses, prior systemic therapy, and gender were found to have no significant impact on the survival of SKCM (P > 0.05) (**Figure 5A**). Multivariate Cox analysis identified N stage, T stage, and risk score as independent prognostic factors (P < 0.05) (**Figure 5B**).

A nomogram incorporating T stage, N stage, and risk score was constructed to reliably predict the prognosis of patients with SKCM (**Figures 5C, D**).

### 3.5 Genes in high and low-risk cohorts were mainly related to cytokine-mediated cellular immune responses

**Figure 6:**
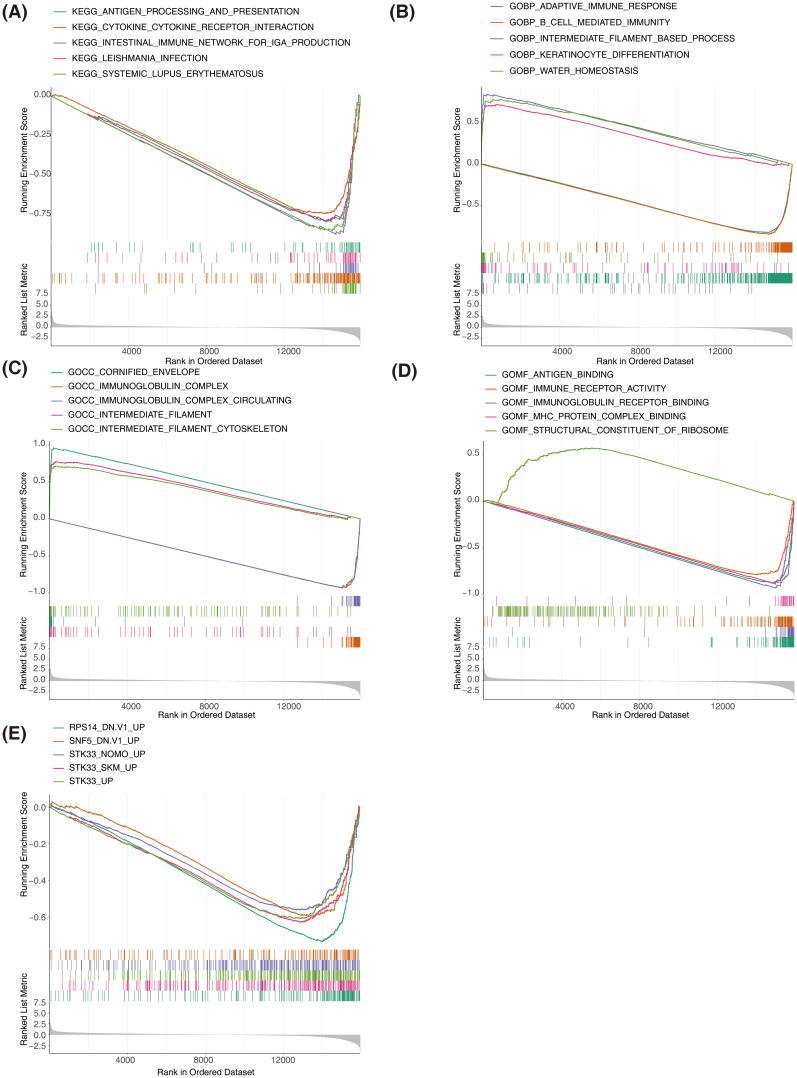
Gene set enrichment analysis (GSEA) of high-risk and low-risk groups in SKCM. **(A)** GSEA of KEGG pathways showing significant enrichment in the high-risk and low-risk groups. **(B)** GSEA of Gene Ontology Biological Process terms showing significant enrichment in the high-risk and low-risk groups. **(C)** GSEA of Gene Ontology Cellular Component terms showing significant enrichment in the high-risk and low-risk groups. **(D)** GSEA of Gene Ontology Molecular Function terms showing significant enrichment in the high-risk and low-risk groups. The top enriched terms include antigen binding, immune receptor activity, and MHC protein complex binding. **(E)** GSEA of oncogenic signatures showing significant enrichment in the high-risk and low-risk groups.

KEGG analysis results indicated that genes in both groups were predominantly associated with interactions between cytokines and cytokine receptors, intestinal immunity networks for IGA production, systemic lupus erythematosus, and allogeneic transplant rejection, among others (**Figure 6A**). GO biological process analysis revealed substantial enrichment of genes in both groups in processes such as adaptive immune response, epidermal and keratinocyte differentiation, and biological processes involving intermediate filaments (**Figure 6B**). GO cellular component analysis showed significant enrichment in components like the cornified envelope, immunoglobulin complex, and intermediate filaments (**Figure 6C**). GO molecular function analysis identified key functions such as antigen binding, immune receptor activity, immunoglobulin receptor binding, and MHC protein complex binding (**Figure 6D**). According to the oncogenic signature database, GSEA highlighted a primary association with the upregulation of *RPS14, SNF5*, and *STK33* (**Figure 6E**).The above results established associations between SKCM prognostic genes and key biological processes such as immune regulation, cell differentiation, and oncogenic pathways through systematic functional genomics analysis. This not only provided a logical framework for explaining the molecular mechanisms of prognostic differences but also laid a data foundation for developing combined targets for immunotherapy.

### 3.6 Nine types of differential immune cells existed between high- and low-risk groups, and prognostic genes were correlated with most immune cells

**Figure 7:**
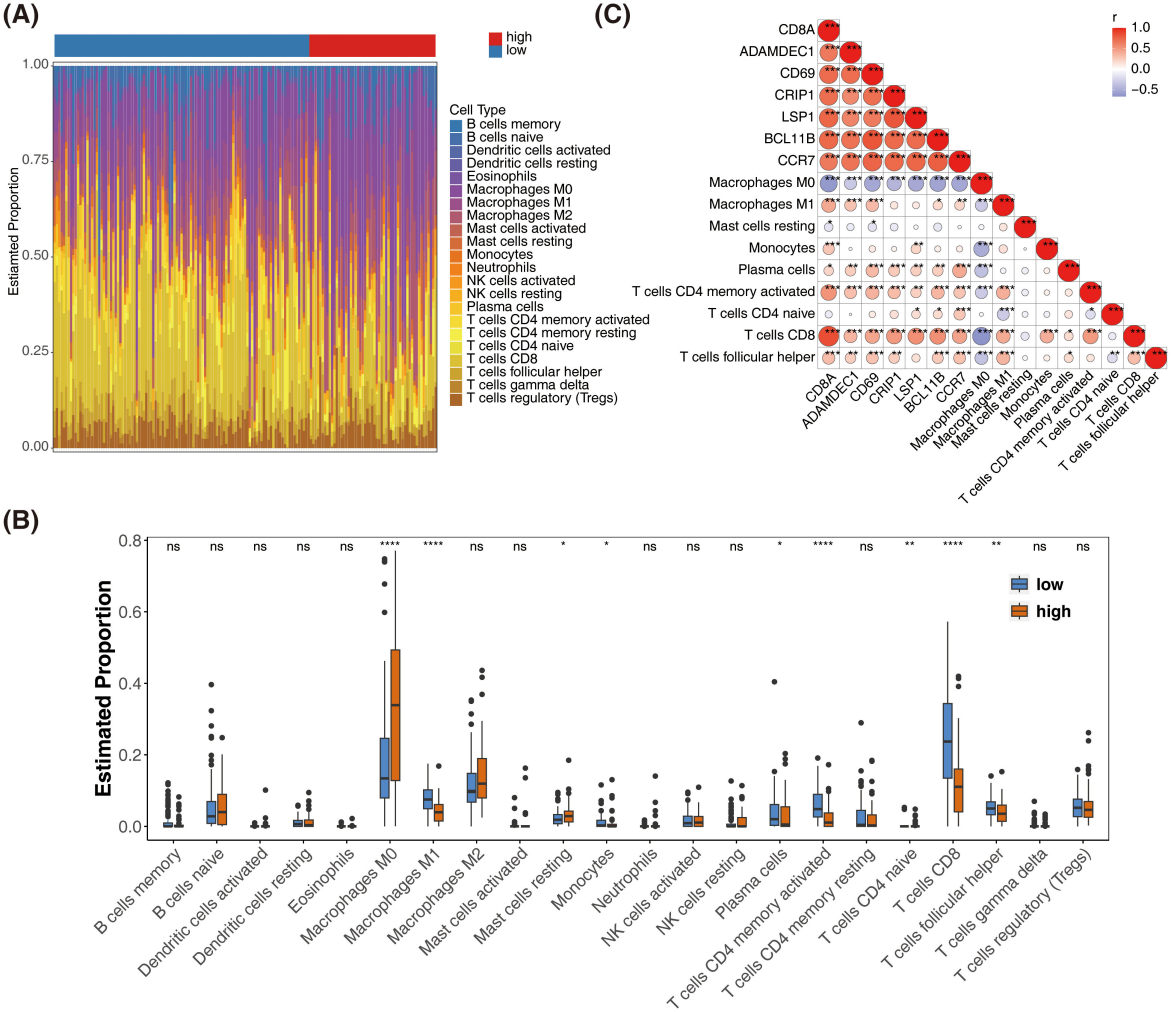
Immune cell infiltration analysis and correlation with prognostic genes in SKCM. **(A)** Bar plot showing the distribution of 22 immune cell types in high-risk and low-risk groups. **(B)** Box plots comparing the estimated proportions of significantly different immune cell types between high-risk and low-risk groups. *represented p < 0.05,** represented p < 0.01, and *** represented p < 0.001. **(C)** Heatmap showing the correlation between prognostic genes and differentially infiltrated immune cells. The color scale represents the correlation coefficient, with red indicating positive correlation and blue indicating negative correlation.* represented p < 0.05, ** indicated p < 0.01, **** denoted p < 0.0001, and ns signified no significant difference.

The distribution of immune cells was analyzed in samples from the high- and low-risk groups (**Figure 7A**). Significant differences were observed in the distribution of nine immune cell types between the two groups (**Figure 7B**). Prognostic genes showed an inverse correlation with Macrophage M0 and a positive correlation with most differentially infiltrating immune cells (**Figure 7C**). Through immune cell infiltration analysis, the dynamic associations between SKCM prognostic genes and the tumor immune microenvironment were revealed, providing key insights for deciphering the immunological mechanisms of prognostic differences, developing immunotherapy biomarkers, and optimizing combination treatment strategies.

### 3.7 Prognostic genes-specific ceRNA network: regulation of CD69, LSP1, and BCL11B by miRNAs and lncRNAs

**Figure 8:**
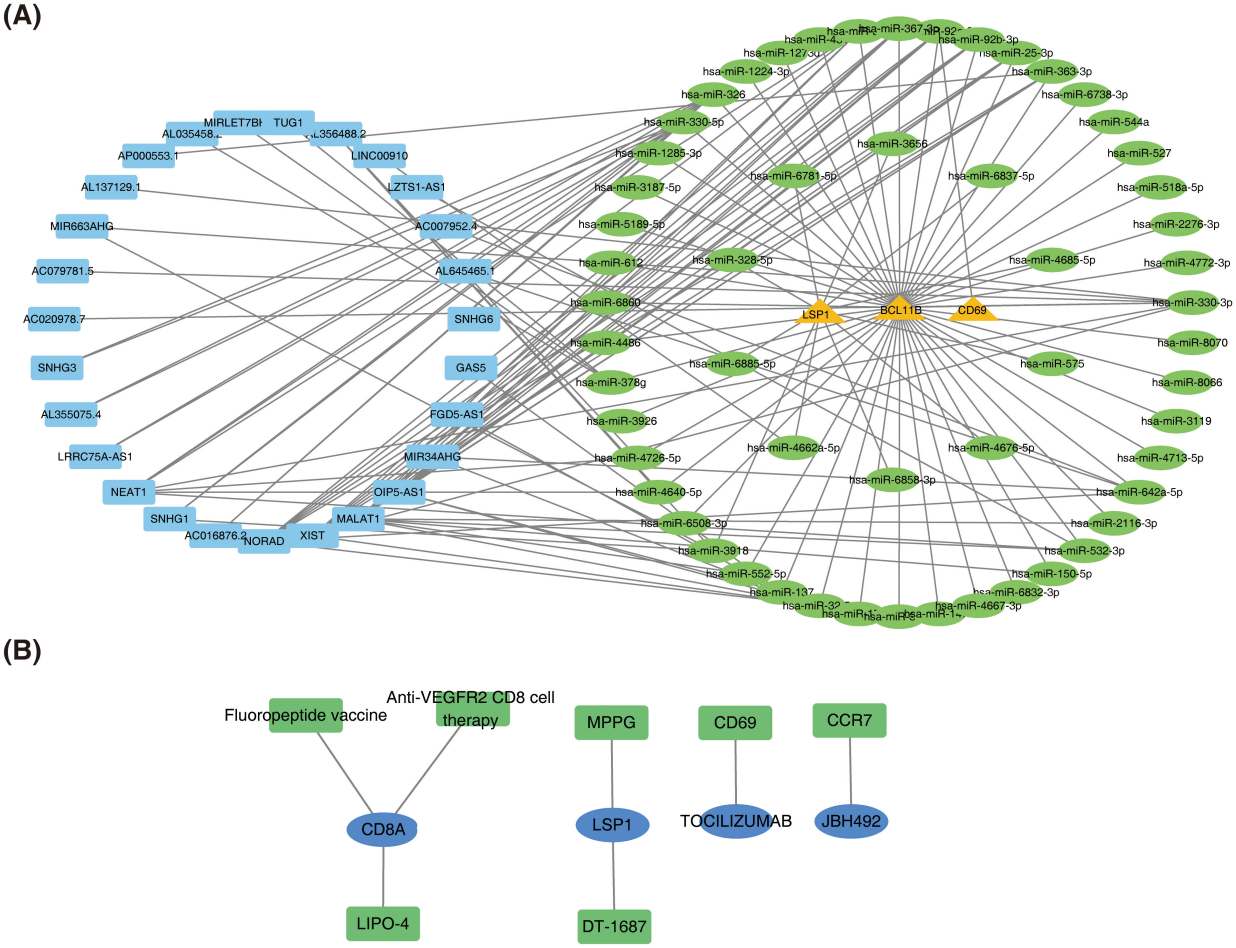
Construction of the ceRNA network and prediction of potential therapeutic agents for SKCM. **(A)** Competing endogenous RNA (ceRNA) network. **(B)** Network of potential therapeutic agents targeting prognostic genes.

miRNA prediction was performed for three prognostic genes (*CD69*, *LSP1*, and *BCL11B*), leading to the construction of a ceRNA network comprising three mRNAs, 56 miRNAs, and 27 lncRNAs (**Figure 8A**). Among these, *SNHG3*, *AL355075*.4, and *LRRC75A-AS1* regulate *BCL11B* expression by modulating hsa-miR-326 and hsa-miR-330-5p. The non-coding RNA regulatory mechanisms of SKCM prognostic genes were revealed above, and potential ceRNA regulatory axes and therapeutic targets were screened out, providing a theoretical basis for deepening the understanding of tumor molecular mechanisms and developing novel biomarkers or RNA therapies.

### 3.8 A total of seven potential therapeutic agents for SKCM based on prognostic genes CD8A, LSP1, CD69, and CCR7

Seven potential therapeutic agents for SKCM were identified based on *CD8A*, *LSP1*, *CD69*, and *CCR7*, while no agents were predicted based on *ADAMDEC1, CRIP1, or BCL11B* (**Figure 8B**). The identified therapeutic agents included Fluoropeptide vaccine, *LIPO-4*, *Anti-VEGFR2 CD8 cell therapy*, *MPPG*, *DT-1687*, *TOCILIZUMAB*, and *JBH492*. By integrating prognostic gene expression and drug sensitivity data, the screening range of therapeutic agents for SKCM was narrowed, providing candidate regimens for personalized treatment.

### 3.9 CD8A, ADAMDEC1, CD69 and CRIP1 were down-regulation in SKCM cell lines, while BCL11B was up-regulation

**Figure 9:**
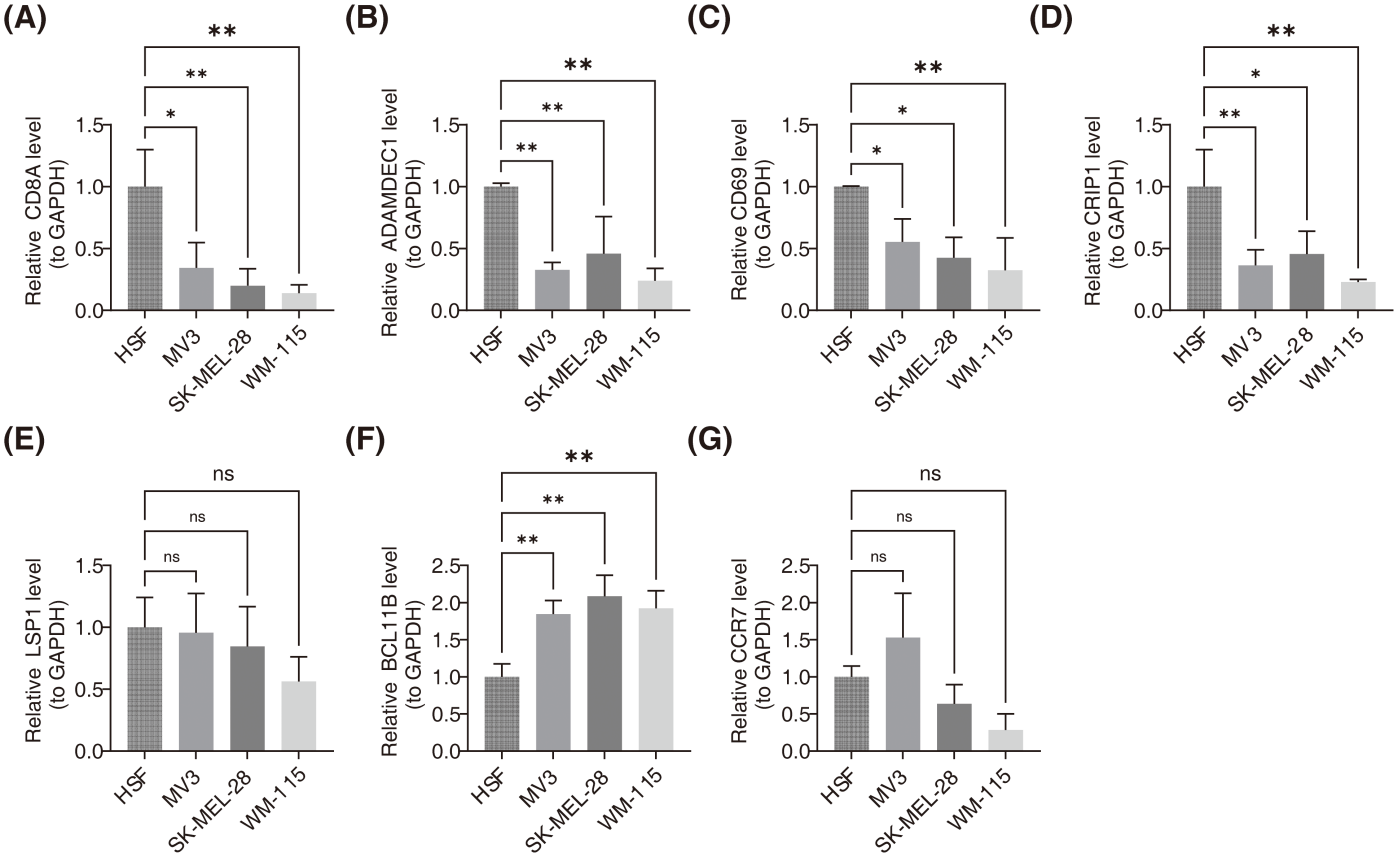
Validation of prognostic gene expression levels in SKCM cell lines using RT-qPCR. **(A)** Relative expression levels of CD8A in HSF, MV3, SK-MEL-28, and WM-115 cell lines. ** indicated p<0.01. **(B)** Relative expression levels of ADAMDEC1 in HSF, MV3, SK-MEL-28, and WM-115 cell lines. ** indicated p<0.01 **(C)** Relative expression levels of CD69 in HSF, MV3, SK-MEL-28, and WM-115 cell lines. ns signified no significant difference. **(D)** Relative expression levels of CRIP1 in HSF, MV3, SK-MEL-28, and WM-115 cell lines. * represented p<0.05, ** indicated p<0.01. **(E)** Relative expression levels of LSP1 in HSF, MV3, SK-MEL-28, and WM-115 cell lines. * represented p<0.05, ** indicated p<0.01. **(F)** Relative expression levels of BCL11B in HSF, MV3, SK-MEL-28, and WM-115 cell lines. * represented p<0.05, ** indicated p<0.01. **(G)** Relative expression levels of CCR7 in HSF, MV3, SK-MEL-28, and WM-115 cell lines. ns signified no significant difference.

As presented in **Figure 9**, RT-qPCR analysis demonstrated that in SKCM cell lines (*MV3*, *SK-MEL-28*, and *WM-115*), expression levels of *CD8A*, *ADAMDEC1*, *CD69*, and *CRIP1* were significantly lower compared to the HSF cell line, while *BCL11B* expression was significantly higher. No significant difference in the expression of *LSP1* and *CCR7* was observed.

## 4 Discussion

Prognostic prediction in SKCM is essential for effective diagnosis and treatment. PANoptosis-related genes have been shown to predict OS in several tumors.^13,14^ In this study, a novel prognostic model for SKCM was developed using bioinformatics approaches, aiming to provide a theoretical foundation for its treatment.

A total of 26 DEPRGs were identified, which were primarily involved in cytokine and chemokine-mediated immune responses, as revealed by GO and KEGG pathway enrichment analysis. Previous studies have indicated that high expression of certain cytokines/chemokines (e.g., *APRIL/CXCL10/CXCL13*) in tumors correlates with improved OS (36). From these findings, seven prognostic genes—*CD8A*, *ADAMDEC1*, *CD69*, *CRIP1*, *LSP1*, *BCL11B*, and *CCR7*—were identified as independent prognostic factors. The results of the multicollinearity test showed that the VIF values of each genes was less than 5, indicating there was no significant multicollinearity issue among the prognostic genes in the model. This suggests that each prognostic gene can independently affect the prognosis of SKCM patients, allowing the model to accurately evaluate the contribution of individual genes in prognosis prediction. These findings ensure the stability and reliability of the established multivariate Cox regression model (37). A prognostic model was then constructed incorporating these genes along with N and T stages. *CD8A*, a glycoprotein primarily expressed on cytotoxic T lymphocytes, which cleave the cytoskeleton and nuclear chromatin, thereby inducing tumor cells apoptosis (38). Our results confirmed that *CD8A* showed the strongest positive correlation with *CD8^+^* T cells, aligning with previous reports (39). ADAMDEC1, as a member of the metalloproteinase family, can cleave Gasdermin family proteins, release their N-terminal domains, form pores on the cell membrane, cause the release of intracellular contents and trigger inflammatory responses, which drive the tumor microenvironment toward a pro-inflammatory state (40). CD69, as an early activation marker, modulates calcium ion signaling in T cells to influence their tumoricidal activity, and also participates in the regulation of inflammatory cytokine release, thereby indirectly affecting the dynamic balance of the PANoptosis pathway. The ceRNA-based CD69 axis has emerged as a promising biomarker for the diagnosis and prognosis of SKCM (41, 42).

*LSP1* is expressed across various immune cells, including lymphocytes, neutrophils, and macrophages (43). *LSP1* also activates the RIPK3-MLKL signaling axis, promoting MLKL phosphorylation and translocation to the cell membrane, thereby inducing necroptosis and releasing damage-associated molecular patterns such as HMGB1, which further activates innate immune cells and reshapes the tumor microenvironment (37, 44). *CRIP1* and *BCL11B* are involved in intestinal zinc transport and are closely linked to B-cell malignancies (45). CRIP1 may regulate the assembly and activation of the NLRP3 inflammasome by interacting with pyroptosis-related adaptor proteins, thereby indirectly influencing the pyroptosis process. Abnormal expression of CRIP1 could potentially exacerbate the inflammatory cascade in SKCM (46). BCL11B inhibits caspase-8 activity to block extrinsic apoptosis signaling transduction, consequently enabling tumor cells to evade immune clearance (47). CCR7, expressed in various lymphoid tissues, activates B and T lymphocytes and facilitates tumor cell migration by mediating interactions between tumor cells and chemokines CCL19/CCL21, and may enhance tumor cell anti-apoptotic capacity through regulation of necroptosis-related gene expression (48). These genes do not function in isolation within the PANoptosis regulatory network, but rather form a multi-level regulatory framework through complex cross-talk between upstream/downstream signaling and protein interactions. Furthermore, these prognostic genes participate in most immune-related receptors and biological functions, indicating that SKCM prognosis is closely related to the immune system.

A risk model was constructed based on prognostic genes, and a prognostic model was further developed by integrating N stage and T stage. While existing SKCM prognostic models predominantly focus on traditional apoptosis-related genes, immune checkpoints, or metabolic pathway-related genes (49), our study pioneers the incorporation of PANoptosis, a novel biological process, into prognostic evaluation. By screening prognostic genes closely associated with PANoptosis, our model not only incorporates conventional factors like TN stage but also elucidates the mechanism of tumor progression from the perspective of inflammatory programmed cell death (50). In terms of predictive performance, our model maintained AUC values ranging from 0.625 to 0.681 for the 1–5 year ROC curves in different datasets. Although it did not reach extremely high predictive accuracy, the model exhibited consistent performance across cohorts and improved generalizability compared to previous models developed based on a single dataset (51). The nomogram constructed in this study, integrating T stage, N stage, and risk score, provides clinicians with a visual and individualized prognosis prediction tool. Compared with previous models that solely relied on clinical stage or a single gene, it can more comprehensively assess patient risk (51, 52).

Further analysis revealed the association of prognostic genes with SKCM progression. *CD8A* has been reported to influence SKCM progression by regulating immune infiltration or immune escape. Moreover, *CD8A* is closely linked to immunotherapy response and serves as a predictive therapeutic biomarker. This can help optimize anti-PD-1 therapy, improving patient outcomes (53). *ADAMDEC1* plays a pivotal role in immune escape and regulating the tumor microenvironment in SKCM, particularly affecting immune-related gene expression. As a potential tumor antigen, *ADAMDEC1* may serve as a target for mRNA vaccines, with patients exhibiting immune subtype IS2 potentially more responsive to these vaccines (54). The ceRNA network regulates gene expression through complex interactions by competitively binding miRNAs via RNA molecules, thereby departing from traditional linear regulatory models (55). *CD69* interacts with non-coding RNAs such as *OIP5-AS1* and *MALAT1* through the ceRNA network, influencing the immune microenvironment and response. Furthermore, in the ceRNA network, lncRNAs such as SNHG3, AL355075.4, and LRRC75A-AS1 may indirectly regulate BCL11B expression by adsorbing hsa-miR-326 and hsa-miR-330-5p, suggesting their potential central regulatory role in SKCM progression (56, 57). *BCL11B* has been closely linked to SKCM progression and treatment, particularly in melanoma brain metastases. Melanoma-specific gene regulatory networks have identified *BCL11B* as a potential therapeutic target, offering new strategies for personalized immunotherapy and targeted treatments (58).

Additionally, significant differences were observed in the infiltration levels of nine immune cell types when assessing the immune landscape of patients with SKCM exhibiting varying risk profiles. Prognostic genes showed an inverse correlation with Macrophage M0 and a positive correlation with most differentially infiltrating immune cells. However, the specific underlying mechanisms of these interactions remain to be explored in greater depth. Furthermore, potential therapeutic strategies for SKCM were explored. Fluoropeptides may enhance immune response capabilities, while lipopeptide vaccines could trigger sustained *CD4^+^* and *CD8^+^* T-cell responses (59, 60). VEGFR2-targeting agents have been shown to promote osteopontin secretion by *CD8^+^* T cells and facilitate robust immune cell infiltration and activation (61). *Tocilizumab* may control cytokine release syndrome in the context of immunotherapy (62).JBH492 may affect the biological behavior of SKCM cells or the tumor microenvironment by targeting and regulating genes such as CD8A and LSP1. However, the specific mechanism still requires further exploration (63).

Finally, RT-qPCR confirmed the mRNA expression levels of the seven prognostic genes. The mRNA levels of *CD8A*, *ADAMDEC1*, *CD69*, and *CRIP1* were significantly downregulated, while *BCL11B* showed significant upregulation. These findings suggest that these genes could serve as biomarkers for SKCM diagnosis and treatment, regardless of gender or lesion type. Consistent with our results, a previous study reported increased mutation frequencies of *FAM135B* and downregulation of genes such as *CD8A*, *GBP5*, *KIAA0040*, and *SAMHD1* in the iC3 subtype, which is associated with the most aggressive SKCM cases, highlighting the critical role these genes play in tumor progression and immune response (64). Another study found that *CD69* could serve as a prognostic marker for SKCM, influencing immune response through interactions with genes like *PTPRC* and *IL7R*, and impacting tumor progression by regulating the tumor immune microenvironment (55). No significant differences in the expression of *LSP1* and *CCR7* were observed between the two groups, which may be attributed to the small sample size or biological variations. These findings will be validated through additional experiments.

In conclusion, this study identified PANoptosis-related genes associated with SKCM prognosis and explored their potential roles in SKCM treatment. However, this study has some limitations. In terms of validation, it primarily relied on bioinformatics analysis and small-sample RT-qPCR experiments, lacking functional validation *in vitro* and *in vivo* such as animal models or cellular functional experiments, and failing to clarify the causal role of identified genes in SKCM progression. Besides, there is a lack of longitudinal data on patients’ long-term follow-up, making it impossible to dynamically track disease progression in relation to gene expression changes. Regarding data limitations, the datasets used lacked key information including BRAF/NRAS gene mutation status and contained insufficient ulcer samples, affecting the comprehensiveness of the analysis. Missing clinical variables also restricted the clinical applicability of the model. Additionally, the PANoptosis score system exhibits shortcomings due to its limited gene selection, only including 19 genes, which fails to fully capture the complexity of PANoptosis biology. Besides, the subjective median-based grouping method compromises conclusion generalizability. In the future, the clinical value of prognostic genes will be verified through multicenter studies by collaborating with clinical institutions to expand sample collection including tissues and blood, and incorporate complete follow-up data. The association between gene expression and disease progression at the protein level well also be validated using immunohistochemistry and Western blot. In exploring functional mechanisms, we will construct cell models with gene knockdown/overexpression and *in vivo* mouse models, combined with experiments on proliferation, migration, and apoptosis, to elucidate the role of prognostic PANoptosis-related genes in SKCM progression. Multi-source data integration will be enhanced by incorporating clinical variables such as BRAF/NRAS mutation status and ulceration characteristics to improve the model variable system. In the optimization of PANoptosis scoring, we will expand gene screening range and replace median-based grouping with scientific cluster methods to achieve precise patient stratification. Concurrently, rigorous pharmacological experiments will be designed to validate therapeutic effects of predicted drugs on SKCM through cell proliferation inhibition and apoptosis induction assays, thereby facilitating the research toward clinical application.

## Data Availability

The original contributions presented in the study are included in the article/**Supplementary Material**. Further inquiries can be directed to the corresponding authors.
