# Supplementary material for: The role and prognostic value of PANoptosis-related genes in skin cutaneous melanoma
Source: Front Immunol. 2025 Jun 6;16:1605977. doi: 10.3389/fimmu.2025.1605977 (PMC12179157; doi:10.3389/fimmu.2025.1605977)
Supplement: Supplementary file 1 [file SupplementaryFile1.pdf]

Supplementary Material

1 Supplementary Data

Supplementary Figure 1. The results of the multicollinearity test for the prognostic genes.

Supplementary Table 1. 19 PANoptosis-related genes.

Supplementary Table 2. RT-qPCR reaction system.

Supplementary Table 3. Primer sequences used for RT-qPCR.

Supplementary Table 4. RT-qPCR amplification conditions.

2 Supplementary Figures and Tables

Supplementary Figure 1. The results of the multicollinearity test for the prognostic genes

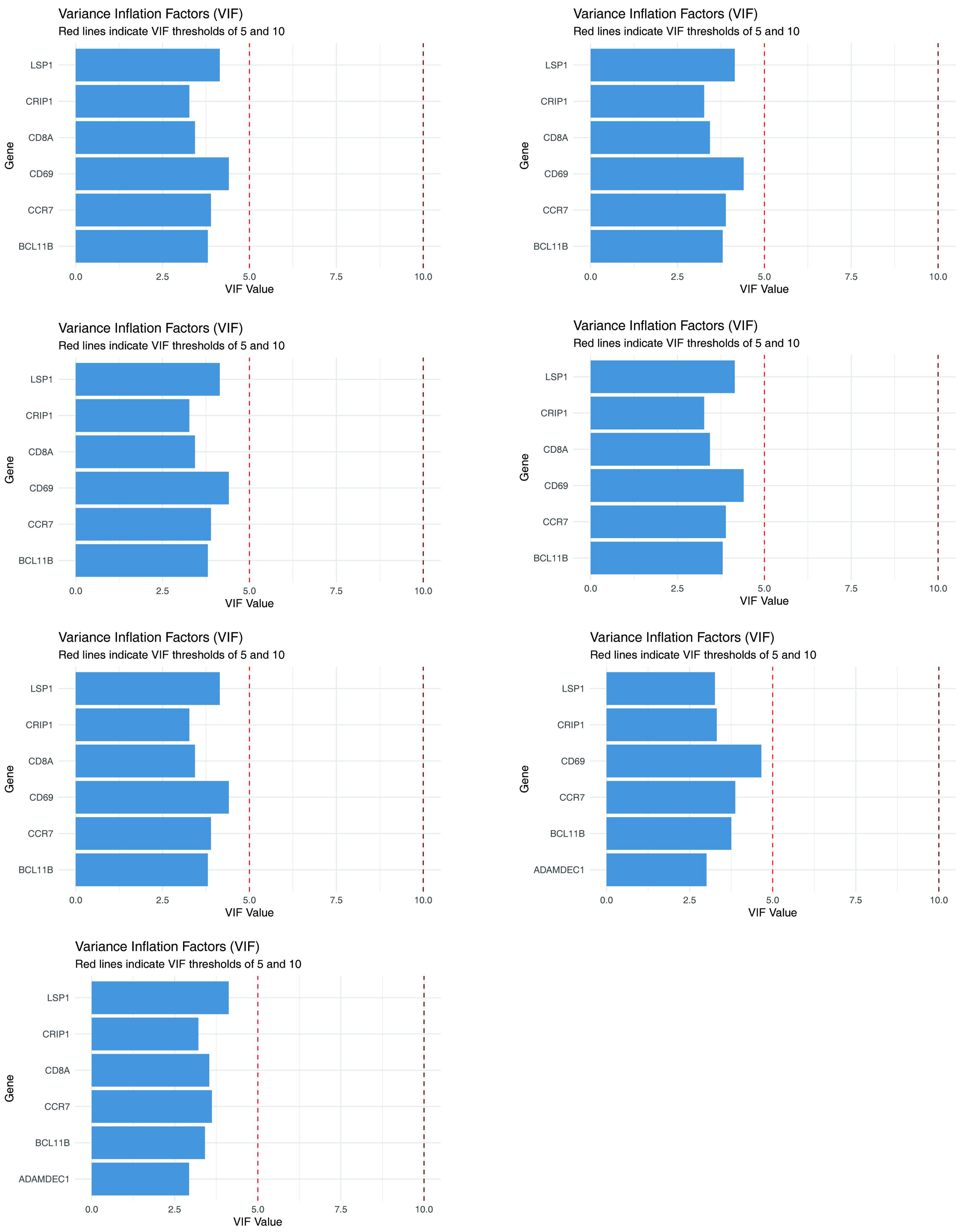

Supplementary Table 1. **19 PANoptosis-related genes.**

Gene	Type

CASP8	PANoptosis
FADD	PANoptosis
CASP6	PANoptosis
NLRP3	PANoptosis
TAB2	PANoptosis
TAB3	PANoptosis
PSTPIP2	PANoptosis
TNFAIP3	PANoptosis
CASP7	PANoptosis
PARP1	PANoptosis
GSDMD	PANoptosis
MLKL	PANoptosis
IRF1	PANoptosis
AIM2	PANoptosis
ZBP1	PANoptosis
CASP1	PANoptosis
RIPK1	PANoptosis
RIPK3	PANoptosis
TRADD	PANoptosis

Supplementary Table 2. RT-qPCR reaction system.

Component	Volume
cDNA	3ul
2xUniversal Blue SYBR Green qPCR Master Mix	5ul
Forward primer (10μM)	1ul
Reverse primer (10μM)	1ul

Supplementary Table 3. Primer sequences used for RT-qPCR.

Primer	Sequence
CD8A F	GACTTGTGGGGTCCTTCTCC
CD8A R	CTGGGCTTGTCTCCCGATTT
ADAMDEC 1 F	GCCATGTCCTTGGTATGCCT
ADAMDEC 1 R	CGTTAGGGCTTCACAGCAGA
CD69 F	GATGCCACCAGTCCCCATTT
CD69 R	GTAGCCAACCCAGTCCTCAG
CRIP1 F	AAACCCTACTGCAACCACCC
CRIP1 R	ATTAGGGGCAACAAGGGAGC
LSP1 F	GTATCAGGGCTCCTTGGTCCC
LSP1 R	CGTGCTGTGCATATGGCTTT
BCL11B F	GCCATAGAGAGACCGAGAGC
BCL11B R	GAGGCTCCTTCCCAGTTCAC
CCR7 F	AGCGTTGAACCGTGAAGAGT

CCR7 R	GCATTTGTAGTCCTGCTGCG
Reference gene-GAPDH F	CGAAGGTGGAGTCAACGGATTT
Reference gene-GAPDH R	ATGGGTGGAATCATATTGGAAC

Supplementary Table 4. RT-qPCR amplification conditions.

	Temperature	Time
Initial denaturation	95℃	1min
Denaturation	95℃	20s
Annealing	55℃	20s
Extension	72℃	30s
